# Nitrous Oxide as an Anæsthetic

**Published:** 1866-10

**Authors:** 


### Editorial.

### NITROUS OXIDE AS AN ANÆSTHETIC.

We have had many inquiries recently in reference to nitrous oxide as an anæsthetic, more particularly with reference to its practicability in dental practice. There is on the part of every progressive member of the profession a desire, and properly so, to avail himself of every new thing in the use of which great advantage is to be derived, or is even anticipated. This is not, in one sense, a new thing, though the demonstration of its practicability is of recent occurrence. From the limited experience we have had with this agent, and from all the information we have been able to gather in reference to it, we have no hesitancy in expressing the full belief that it is the safest general anæsthetic in use. We are not aware of a well authenticated case of death by it. Its mode of action upon the system is very different from that of chloroform or ether; it increases, while they diminish, the amount of oxygen in the blood. We are free to admit that injury may be occasioned by its injudicious or excessive administration, but there is certainly not the same liability to sudden fatal results. It should ever be remembered that any agent that acts as rapidly and as extensively as this, is liable, especially in the hands of a careless or ignorant operator, to do mischief. It is also important that the agent be pure.

It has advantages in the following respects: its comparative safety, its rapidity of action, and its almost universal efficiency. We have scarcely heard of a case in which it did not act promptly and well. It is especially adapted for brief minor operations. It may be equally well employed by both the general surgeon and the dentist. The continuance of its influence is brief, ranging from fifteen seconds to a minute and a half, under one administration, so that where much is to be done, it necessarily involves rapid execution or repeated administration; and for operations in the mouth, where there is much hemorrhage, the latter is objectionable.

Another difficulty that attaches to the use of this agent is that it cannot be manufactured and kept so conveniently and in so small a compass as chloroform or ether. The present mode of administration is cumbersome and unsightly, though we believe some improvement has recently been made in this particular, by the substitution of a large rubber tube to the general gas-holder, instead of the inhaling bag. Great care should be exercised in the selection of the material from which to prepare the gas, and also in the manner of its preparation. No one should venture its preparation who is not a good chemist, or who has not been trained by a good chemist in this process.

We do not regard the use of nitrous oxide as an anæsthetic practicable in the hands of the ordinary dental practitioners for daily use. It would thus consume too much time, and divert the attention too much; but it can be made very valuable and highly advantageous by establishing in all cities large enough to sustain such an enterprise an office or offices, where special attention shall be devoted to its administration. Such arrangements are already in operation at a few points, and operate admirably.

In smaller towns and in villages it may be made practicable by the dentist giving his exclusive attention to that particular subject at fixed periods: for instance, one day in the week to extract teeth, and for the performance of such other operations as would require the gas.

For directions for its preparation and administration, see a little work prepared by Prof. Barker, of Philadelphia, and noticed on another page of this number; or a little pamphlet prepared by Dr. A. M. Leslie, of St. Louis, in which very brief and concise directions are given.

Apparatus for the manufacture of nitrous oxide gas, for anæsthetic purposes, is manufactured by several parties in this country. Dr. A. M. Leslie, of St. Louis, makes the apparatus in two or or three forms; one to be put up as a permanent fixture, another put up in very small compass, that he who runs may take it with him. These, we think, are sold at from $25 to $45 each. They are also sold by James Leslie, of this city.

The apparatus manufactured by G. W. Sprague, of Boston, is in some respects the most perfect yet produced; it is self-regulating in regard to heat, which is a very important item; but we believe this feature can only be employed where the common illuminating gas is used. It is a very fine apparatus, indeed, as we know by practical test. The cost is about $100.

